# Doublecortin-like kinase 1 promotes fibroblast activation and fibrotic progression through Smad3 binding in idiopathic pulmonary fibrosis

**DOI:** 10.1186/s12929-026-01258-7

**Published:** 2026-05-22

**Authors:** Lee-Yuan Lin, Wun-Hao Cheng, Yu-Chih Wu, Heng-Ching Wen, Hsao-Hsun Hsu, Chia-Hao Liu, Jing-Yun Chen, Wen-Shan Chang, Ying-Jung Wu, Jie-Syuan Wu, Fara Silvia Yuliani, Chueh-Yi Wang, Chien-Huang Lin, Bing-Chang Chen

**Affiliations:** 1 Graduate Institute of Medical Sciences, College of Medicine, Taipei Medical University, No. 250, Wuxing St., Xinyi Dist., Taipei, 110 Taiwan; 2 School of Medicine, College of Medicine, Taipei Medical University, Taipei, Taiwan; 3 School of Respiratory Therapy, College of Medicine, Taipei Medical University, No. 250, Wuxing St., Xinyi Dist., Taipei, 110 Taiwan; 4 National Taiwan University Hospital & College of Medicine, National Taiwan University, Taipei, Taiwan; 5 Department of Surgical Oncology, National Taiwan University Cancer Center, Taipei, Taiwan; 6 Department of Pharmacology and Therapy, Faculty of Medicine, Public Health, and Nursing, Universitas Gadjah Mada, Yogyakarta, Indonesia; 7 Laboratory Animal Center, Taipei Medical University, Taipei, Taiwan; 8 Chen Wei-Tien Research Center of Thoracic Medicine, Taipei Medical University, Taipei, Taiwan

**Keywords:** DCLK1, Idiopathic Pulmonary Fibrosis, TGF-β, Smad3, Fibroblasts

## Abstract

**Background:**

Idiopathic pulmonary fibrosis (IPF) is a progressive, fatal lung disease with limited treatment options. Although the results of a gene expression analysis revealed that lung tissues in individuals with IPF contain high levels of doublecortin-like kinase 1 (DCLK1), the role of DCLK1 in fibroblast activation remains unclear. The present study examined the function of DCLK1 in IPF and bleomycin-induced pulmonary fibrosis, exploring its potential as a therapeutic target warranting further investigation.

**Methods:**

We investigated the role of DCLK1 in fibroblast activation and pulmonary fibrosis in lung tissues from patients with IPF, a bleomycin-induced pulmonary fibrosis mouse model, and cultured normal human lung fibroblasts. DCLK1 knockout mice and mice treated with the selective DCLK1 inhibitor DCLK1-IN-1 were used to evaluate the effects of genetic DCLK1 deficiency and pharmacological DCLK1 inhibition on fibrotic progression and lung function.

**Results:**

DCLK1 was markedly upregulated in the IPF lung tissues and in bleomycin-induced fibrotic lung tissues. Global deletion of *Dclk1* considerably attenuated fibrotic remodeling and preserved lung function in mice. In addition, transforming growth factor β (TGF-β) induced DCLK1 expression in normal human lung fibroblasts through Smad3 and NF-κB signaling, while Akt/DCLK1/Smad3 signaling was associated with fibroblast activation and profibrotic marker expression. Moreover, DCLK1 was associated with Smad3, and these findings were consistent with DCLK1–Smad3-associated signaling linked to connective tissue growth factor expression. Finally, oral administration of DCLK1-IN-1 slowed fibrotic progression and preserved lung function in bleomycin-treated mice.

**Conclusions:**

DCLK1 is associated with fibroblast activation and pulmonary fibrosis, with findings consistent with DCLK1–Smad3-associated signaling linked to profibrotic marker expression. Genetic deletion or pharmacological inhibition of DCLK1 attenuated fibrotic progression and preserved lung function, suggesting that DCLK1 warrants further investigation as a potential therapeutic target in pulmonary fibrosis.

## Background

Idiopathic pulmonary fibrosis (IPF) is a rare disease with an incidence of fewer than 10 cases per million person-years and a prevalence of approximately 40 cases per million [1]. The majority of individuals with IPF survive from 3 to 5 years after they receive a diagnosis [2]. Common symptoms of IPF include cough, dyspnea, and reduced quality of life [3]. In addition, fibrosis-mediated thickening of the pulmonary interstitium reduces the efficiency of gas exchange in the alveoli, hindering diffusing capacity for carbon monoxide [4]. The pathogenesis of IPF involves repetitive epithelial cell injury and senescence leading to abnormal alveolar repair and excessive myofibroblast deposition in the interstitium, which results in pulmonary fibrosis (PF) [5]. The distinguishing pathological characteristic of IPF is the aberrant activation of fibroblasts, which promotes extracellular matrix deposition of fibronectin, collagen, and fibrotic proteins such as connective tissue growth factor (CTGF) and causes them to differentiate into myofibroblasts that express α-smooth muscle actin (α-SMA) [6].

Doublecortin-like kinase 1 (DCLK1) is a serine/threonine kinase in the calcium/calmodulin-dependent kinase family with two microtubule-binding domains [7]. DCLK1 overexpression occurs in several cancers, specifically, colorectal, pancreatic, gastric, renal, and hepatocellular carcinomas, in which it is correlated with poor prognosis, high recurrence, and metastasis [8]. DCLK1 affects tumor initiation and progression and promotes the epithelial–mesenchymal transition in cancer stem cells [9]. DCLK1 also regulates nuclear factor kappa B (NF-κB) in colorectal and gastric carcinomas, promoting angiogenesis and the epithelial–mesenchymal transition and facilitating metastasis [10]. Moreover, a study has linked DCLK1 to viral sepsis, inflammatory mediator expression, and immune cell dysfunction in SARS-CoV-2 infection, suggesting that because DCLK1 inhibition blocks proinflammatory caspase-1/interleukin-1β signaling in infected cells, it may be applicable in treating COVID-19 [11]. Furthermore, a study reported that DCLK1 expression is markedly enhanced in the alveolar epithelial cells of patients with PF and in mice with bleomycin (BLM)-induced PF [12]. Studies have also indicated that DCLK1 is involved in fibrosis and scarring, and the results of gene expression analyses have suggested that it may be implicated in IPF [13, 14]. Because abnormal repair and fibrosis of alveolar tissue are key characteristics of IPF pathogenesis, elucidating how DCLK1 is regulated in lung fibroblasts may provide insight into potential therapeutic strategies for IPF.

Transforming growth factor beta (TGF-β) is heavily involved in fibrotic signaling in IPF, and lung fibroblasts are the principal target of TGF-β that contributes to fibrogenesis [15, 16]. TGF-β family members and proteases, integrins, and extracellular matrix molecules regulate gene expression through Smad and non-Smad signaling cascades [17]. For example, TGF-β induces collagen expression through the Smad2/3 pathway in IPF [18]. TGF-β activates the PI3K/Akt pathway by directly inducing Akt phosphorylation through PI3K and indirectly upregulating miRNA that inhibits PTEN and Smad7, activating Akt [19]. A study also suggested that DCLK1, a cancer stem cell marker, is associated with the TGF-β signaling pathway, although the mechanism underlying this association is unclear [20], as are the therapeutic effects of inhibiting DCLK1 on PF.

Although studies have suggested that DCLK1 and TGF-β–related fibrotic signaling are correlated, the mechanisms by which DCLK1 regulates fibroblast activation in PF remain poorly understood. In addition, whether DCLK1 participates in TGF-β–Smad signaling to regulate profibrotic gene expression and whether its inhibition mitigates fibrosis in vivo have yet to be determined. Therefore, the present study examined the role of DCLK1 in fibroblast activation and PF and evaluated its potential relevance as a therapeutic target by using complementary genetic and pharmacological approaches in patient-derived samples, cultured lung fibroblasts, and mouse models of BLM-induced fibrosis.

## Materials and methods

### Materials and reagents

Normal human lung fibroblasts (NHLFs) were procured from the American Type Culture Collection (Manassas, VA, USA). Small interfering RNA (siRNA), control siRNA (scRNA), and α-tubulin antibody (T9026) were obtained from Sigma-Aldrich (St. Louis, MO, USA). BLM was purchased from MedChemExpress (Monmouth Junction, NJ, USA). Antibodies for fibronectin (ab2413), α-SMA (ab5694), DCLK1 (ab106635), Smad3 (ab84177), phospho-Smad3 (ab52903), Alexa Fluor-488, Alexa Fluor-555, and Alexa Fluor-647 were sourced from Abcam (Cambridge, MA, USA). Anti-DCLK1 phospho Ser30 (630–620) was procured from ThermoFisher Scientific (Waltham, MA, USA). Anti-CTGF (86,641), Akt (4691), and phospho-Akt (4060) antibodies were obtained from Cell Signaling (Danvers, MA, USA). Anti-fibroblast surface protein (FSP; NB100-1845) was purchased from Novus Biologicals (Littleton, CO, USA**)**. Antirabbit immunoglobulin G (IgG) (sc-2004) and antimouse IgG (sc-2314) were sourced from Santa Cruz Biotechnology (Dallas, TX, USA). A chromatin immunoprecipitation (ChIP) assay kit was acquired from Upstate Biotechnology Millipore (Lake Placid, NY, USA). Lipofectamine 3,000 reagent and minimum essential medium were purchased from Invitrogen Life Technologies (Carlsbad, CA, USA). Fetal bovine serum was sourced from Corning (Corning, NY, USA). A Novolink Max Polymer Detection System was purchased from Leica (Wetzlar, Germany). DCLK1-IN-1 (HY-135985) was purchased from MedChemExpress. Finally, commercially sourced normal lung tissue slides (RS321) were obtained from US Biomax (Derwood, MD, USA).

### Patients and samples

Lung tissue samples were obtained from patients with IPF aged > 20 years who had been evaluated for lung surgery and were undergoing lung transplantation. The control group consisted of 14 samples, including 3 surgically obtained nonfibrotic lung tissue samples and 11 commercially sourced normal lung tissue slides. Surgically obtained control tissues were collected from patients aged > 20 years with lung diseases other than PF or from patients with lung nodules; tumor-free surrounding lung tissue was removed during surgery and used as nonfibrotic control tissue. The exclusion criteria were age < 20 years, pregnancy, and inability to make decisions regarding medical care.

### Study approval

The Research Ethics Committee of National Taiwan University Hospital approved all experiments involving humans (202101098RINC). All animal studies were approved by the Taipei Medical University Institutional Animal Care and Use Committee (LAC2023-0052, LAC2023-0150, LAC2023-0151, and LAC2023-0171). This study also received approval for the use of pathogenic microorganisms for gene recombination research from the Biosafety Committee of Taipei Medical University (G-112-022).

### Single-cell RNA sequencing data processing and analysis

Single-cell RNA sequencing data were retrieved from the Gene Expression Omnibus (GEO) database under accession number GSE136831 [21]. The initial data set contained 312,928 cells across control, IPF, and chronic obstructive pulmonary disorder (COPD) groups. After stringent quality control filters had been applied, cells with between 200 and 6,000 unique features and mitochondrial gene content of < 20% were retained, and the COPD cohort was excluded to yield a final analytical population of 240,915 cells from the control and IPF groups. Data were log-normalized using a scale factor of 10,000 through the Seurat package (version 5.4.0; https://satijalab.org/seurat/). Cell type annotations were assigned on the basis of original study metadata to identify major lineages of endothelial, epithelial, lymphoid, myeloid, and stromal cells, particularly the subset of myofibroblasts. Dimensionality reduction was performed using uniform manifold approximation and projection (UMAP) to visualize spatial comparability across groups. The expression of *DCLK1* was also visualized. Significance between groups was evaluated at the level of single cells by using the Wilcoxon rank-sum test. Because the analysis was hypothesis-driven and limited to the predefined gene DCLK1, raw p values were used, and multiple-testing or false discovery rate correction was not applied.

### Development of a mouse model of knocked out DCLK1

To establish a mouse model of knocked out DCLK1, we crossed mice harboring loxP sites flanking exon 3 of the DCLK1 locus (DCLK1^f/f^) with Pgk1-RFP-Cre/ERT2 transgenic mice, in which Cre recombinase expression is promoted by the *Pgk1* promoter and activated by tamoxifen. Both mouse strains had a C57BL/6 background; DCLK1^f/f^ mice were obtained from the Jackson Laboratory (Bar Harbor, ME, USA), and Pgk1-RFP-Cre/ERT2 mice were obtained from the Rodent Model Resource Center (Taipei, Taiwan). The DCLK1^f/f^ Pgk1-RFP-Cre/ERT2 mice are hereinafter referred to as DCLK1^f/f^/Cre^+^ mice; littermate DCLK1^f/f^ mice lacking the Cre allele served as controls. To induce recombination, tamoxifen (80 mg/kg) was administered through daily intraperitoneal injection for 7 consecutive days, with this followed by twice-weekly administration to enhance and maintain recombination efficiency after BLM or PBS instillation and throughout the experimental period, which resulted in the effective global deletion of the floxed *Dclk1* allele in the Cre-expressing mice. The two mouse genotypes received identical tamoxifen regimens to control for potential tamoxifen-related effects.

### BLM-Induced PF model

PF was induced through intratracheal instillation of BLM (3 mg/kg in phosphate-buffered saline [PBS]) or sham treatment under anesthesia per the procedure described in another study [22]. Tamoxifen-inducible knockout mice and littermate controls received intraperitoneal injections of tamoxifen (80 mg/kg) daily for 7 days before BLM or PBS instillation and twice weekly after instillation until euthanasia [23]. On day 21 or 28 after BLM was instilled, the mice were euthanized through isoflurane inhalation. The right lung was ligated and snap-frozen for protein extraction, and the left lung was inflation-fixed at 25 cm H_2_O with 10% neutral buffered formalin for histological analysis. Blood samples were collected through submandibular venipuncture and centrifuged at 400 × g for 5 min, after which the cell pellets were resuspended in 100 µL of PBS. The concentration of cells in the blood samples was analyzed with a hematology analyzer (ProCyte Dx, IDEXX Laboratories, Westbrook, ME, USA).

### DCLK1-IN-1 oral gavage

To examine pharmacological inhibition in vivo, DCLK1-IN-1 was dissolved in carboxymethyl cellulose solution (0.5 wt% carboxymethyl cellulose, 0.1 wt% Tween 80, and 5 wt% dextran in ddH_2_O). The compound was administered through oral gavage at a dose of 10 mg/kg once daily on the basis of the protocol of an in vivo study that inhibited DCLK1 with acceptable tolerability in murine models [24]. Control mice received an equivalent volume of a vehicle alone. DCLK1-IN-1 treatment was initiated from day 7 after BLM induction and continued daily until harvest.

### Experimental design, randomization, and outcome assessment blinding in murine experiments

Both male and female mice aged 6–8 weeks were used in the inducible DCLK1 knockout experiments, and a comparable sex distribution was maintained across the experimental groups. To minimize sex-related variability in the pharmacological inhibition experiments, only male mice aged 6 weeks were used. Animals were excluded from the analysis only when they died before tissues could be harvested.

In the inducible DCLK1 knockout experiments, experimental mice (DCLK1^f/f^/Cre^+^) and control mice were randomly assigned to intratracheal BLM or PBS groups. In the pharmacological inhibition experiments, the mice were randomly allocated to one of three groups: control, BLM, or BLM plus DCLK1-IN-1 treatment. Randomization was performed before treatment assignment. All investigators performing histological scoring, immunofluorescence quantification, micro computed tomography (microCT) analysis, and lung function measurements were blinded to both the genotype and the treatment group throughout data acquisition and analysis.

### Quantitative evaluation of lung remodeling in PF murine model through in vivo microCT

In vivo microCT imaging of the mice was conducted at the Taipei Medical University Laboratory Animal Center by using an animal microCT system (SkyScan 1176, Bruker, Kontich, Belgium). In this procedure, the mice were anesthetized with 2% isoflurane in oxygen during image acquisition, and scans were conducted without respiratory gating. The following acquisition parameters were used: an X-ray source voltage of 50 kV, source current of 500 μA, aluminum filter of 0.5 mm, exposure time of 87 ms, camera binning of 4 × 4, and an isotropic voxel size of 34.75 μm. Raw projection images were reconstructed using NRecon software (Bruker, Kontich, Belgium) with standard beam-hardening correction and ring-artifact reduction. Lung volumes were isolated and quantitatively analyzed with CTAn software (Bruker, Kontich, Belgium) according to the manufacturer’s protocols. Regions of interest were defined to include lung parenchyma and to exclude major airways and blood vessels.

Density values were used to differentiate normal and fibrotic lung areas, with regions exhibiting densities < − 500 Hounsfield units (HU) classified as normally aerated and functional lung and regions with densities > − 500 HU classified as fibrotic lung tissue on the basis of the protocol of a validated microCT study that established a BLM-induced model of PF [25].

#### Functional assessment of PF progression in mice

The mice were anesthetized through an intraperitoneal injection of Zoletil and Rompun and maintained under deep anesthesia for approximately 10 min. A surgical procedure was performed to open the airway, which was subsequently connected to the FlexiVent system to conduct a lung function assessment. The apparatus was used to control the breathing rate; lung function and airway impedance data were recorded.

#### Detection of DCLK1/Smad3 protein interaction in lung fibroblasts by coimmunoprecipitation

Cells were cultured in 10-cm culture dishes and treated with TGF-β. After the supernatant was removed, whole cell lysates were collected. The quantified protein was subsequently added to DCLK1 antibodies and protein A/G sepharose and reacted for 24 h at 4 °C. The immune precipitate was washed with 500 μL lysis buffer at 4 °C and added to the sodium dodecyl sulfate (SDS) loading buffer. The immunoprecipitated protein was separated using SDS polyacrylamide, transferred to polyvinylidene fluoride (PVDF), and analyzed for binding between DCLK1 and Smad3 antibodies.

#### Detection of nuclear colocalization of DCLK1 and Smad3 by immunofluorescence staining

After the NHLFs on the coverslips had been stimulated with TGF-β for 30 min, the cells were fixed with 4% paraformaldehyde at room temperature for 15 min. Blocking was performed with 5% serum at room temperature for 20 min, with this followed by incubation with specific antibodies at room temperature for 1 h. The coverslips were washed thrice with 0.5% PBS/Tween 20, incubated with Alexa Fluor 488 or Alexa Fluor 594-conjugated secondary antibodies (Molecular Probes, Eugene, OR, USA) for 1 h, and washed thrice. The coverslips were subsequently removed with forceps and mounted with Vectashield (Vector Laboratories, Burlingame, CA, USA) to facilitate fluorescent microscopy.

#### Detection of protein expression in fibrotic lung tissues by histology and immunohistochemistry

Lung tissue sections from mice treated with BLM and PBS were fixed overnight in 4% paraformaldehyde, followed by paraffin embedding and sectioning. Hematoxylin and eosin (H&E) and Masson trichrome staining were performed to assess morphology and collagen expression. To conduct immunohistochemistry (IHC), we deparaffinized tissue sections through immersion in NOVA Histo (Bionovas, Toronto, Ontario, Canada), heated them in a pressure cooker for 10 min, and treated them sequentially with peroxidase and protein block solutions from a Max Polymer Detection System to inactivate them. The sections were incubated with primary antibodies for 30 min, washed thrice with 0.1% tris-buffered saline with Tween-20 (TBST), and subjected to 30-min incubation with secondary antibodies, three TBST washes, and reaction with 3,3′-diaminobenzidine to develop color. After three additional TBST washes, hematoxylin nuclear counterstaining was performed, and the sections were coverslipped. Protein expression changes were analyzed microscopically. Subsequently, three nonoverlapping fields per section were quantified and averaged to generate a single value per sample. Signal intensity was normalized to the number of nuclei within each field. Fields were consistently selected across samples to ensure comparability.

#### NHLF cultures

Primary NHLFs were purchased from the American Type Culture Collection and cultured in modified Eagle medium (MEM) supplemented with 2 mM *L*-glutamine, 0.1 mM nonessential amino acids, 1 mM sodium pyruvate, 10% fetal bovine serum, and antibiotics (10,000 units/mL penicillin, 10,000 μg/mL streptomycin, and 250 μg/mL fungizone) at 37 °C in a 5% CO_2_ incubator in T75 flasks. When the cells had reached approximately 75% confluency, they were harvested by using TrypLE, seeded onto 60-mm dishes, treated with agents, collected, and analyzed using Western blotting.

#### DCLK1 and Smad3 siRNA transfection

NHLFs were cultured in 60 mm dishes at 37 °C in a humidified 5% CO_2_ incubator until approximately 70% confluency was reached. The medium was subsequently replaced with fresh complete medium, and the cells were transfected with siRNA or a nontargeting control siRNA by using Lipofectamine 3,000 in accordance with the manufacturer’s protocol. The siRNAs were mixed with Lipofectamine 3,000 diluted in Opti-MEM and incubated for 20 min at room temperature before being added to the dishes. At 24 h after transfection, the medium was changed to serum-free MEM overnight before TGF-β stimulation. Finally, protein expression and phosphorylation changes were assessed.

#### Dominant negative Akt transfection

NHLFs were cultured in 60-mm dishes at 37 °C in a humidified 5% CO_2_ incubator until approximately 70% confluency was reached. The medium was replaced with fresh complete medium, and the cells were transfected with dominant negative Akt (DN-Akt) or scrambled DNA by using Lipofectamine 3,000 and P3,000 reagents per the manufacturer’s protocol. The DNA was mixed with Lipofectamine 3,000 and P3,000 diluted in Opti-MEM and incubated for 20 min at room temperature before being added to the dishes. At 24 h after transfection, the medium was changed to serum-free MEM overnight before TGF-β stimulation. Finally, protein expression and phosphorylation changes were assessed.

#### Quantification of protein expression in lung and cell lysates through Western blot analysis

At a designated time, the culture medium was removed, and the cells were washed twice with cold PBS and placed on ice. A radioimmunoprecipitation assay lysis buffer (30 μL) was added, and the cells were collected in 1.5-mL centrifuge tubes and centrifuged at 12,000 rpm for 30 min to extract the supernatant. The protein samples were mixed with 5 × Laemmli buffer, heated at 95 °C for 5 min, and subjected to SDS polyacrylamide gel electrophoresis. The separated proteins were transferred to a PVDF membrane, blocked with 5% bovine serum albumin in TBST for 1 h, and washed thrice with TBST for 5 min. Specific antibodies were added and incubated overnight at 4 °C. After three additional 5-min TBST washes, the membrane was incubated with secondary antibodies for 1 h followed by three 10-min TBST washes. An enhanced chemiluminescence solution was applied immediately to detect protein expression.

#### Detection of transcription factor binding to target promoters through ChIP

The cells were fixed with 10% formaldehyde after 30 min of TGF-β stimulation to crosslink transcription factors and chromatin, after which they were collected and subjected to ultrasonic shearing. Immunoprecipitation was performed on the experimental group by using antibodies specific to DCLK1, Smad3, or NF-κΒ p65; the control group was incubated with a rabbit anti-IgG antibody. The precipitated DNA was extracted, and a polymerase chain reaction of the DCLK1, Smad3, and NF-κΒ p65 response elements on the DCLK1 or CTGF promoter regions was performed using specific primers. PCR amplification was performed using cycle numbers within the non-saturated linear amplification range to allow semi-quantitative comparison of band intensities across groups. PCR products were resolved by agarose gel electrophoresis. ChIP-PCR band intensities were quantified by densitometric analysis using the mean band density of each field. For each sample, the ChIP-PCR signal was normalized to the corresponding input DNA signal to account for differences in DNA loading.

The primer sequences were as follows:

Smad3 response element of the CTGF promoter region:

sense: 5′-TCTTAGTTTATATCCAGATA-3′.

antisense: 5′-TTAGTTTTCTCTTAATATATG-3′.

Smad3 response element of the DCLK1 promoter region:

sense: 5′-GCCTCATCTCTCGGCCATAT-3′.

antisense: 5′-TTCTTGGCAGGGCGCACCCA-3′.

NF-κΒ p65 response element of the DCLK1 promoter region:

sense: 5′-GACTGGAGGAAGCTGTGAAC-3′.

antisense: 5′-TGCCTCTTCGGATGACCAA-3′.

#### Data availability

The single-cell RNA sequencing data of IPF and healthy control tissue were obtained from the publicly available GEO data set GSE136831 (https://www.ncbi.nlm.nih.gov/geo/query/acc.cgi?acc=GSE136831) [21]. Additional data are available from the corresponding author upon reasonable request.

#### Statistical analysis

In all in vivo studies, n represents the number of independent animals, whereas in the in vitro experiments, n represents independent biological replicates derived from separate cell cultures. The statistical unit, animal numbers per analysis, number of independent replicates, and statistical tests used are specified in the figure legends.

Values are expressed as means ± standard errors of the mean, and each figure’s sample size is provided. To determine statistical significance, an unpaired Student’s *t* test, a one-way analysis of variance (ANOVA) followed by Dunnett’s test, and a two-way ANOVA followed by Bonferroni’s multiple comparisons test were performed in Prism 7.0a (GraphPad); *p < 0.05 was considered significant in the unpaired Student’s *t* tests, one-way ANOVAs followed by Dunnett’s test, and two-way ANOVAs followed by Bonferroni’s multiple comparisons test.

## Results

### DCLK1 expression and phosphorylation upregulated in lung tissue from patients with IPF

**Fig. 1:**
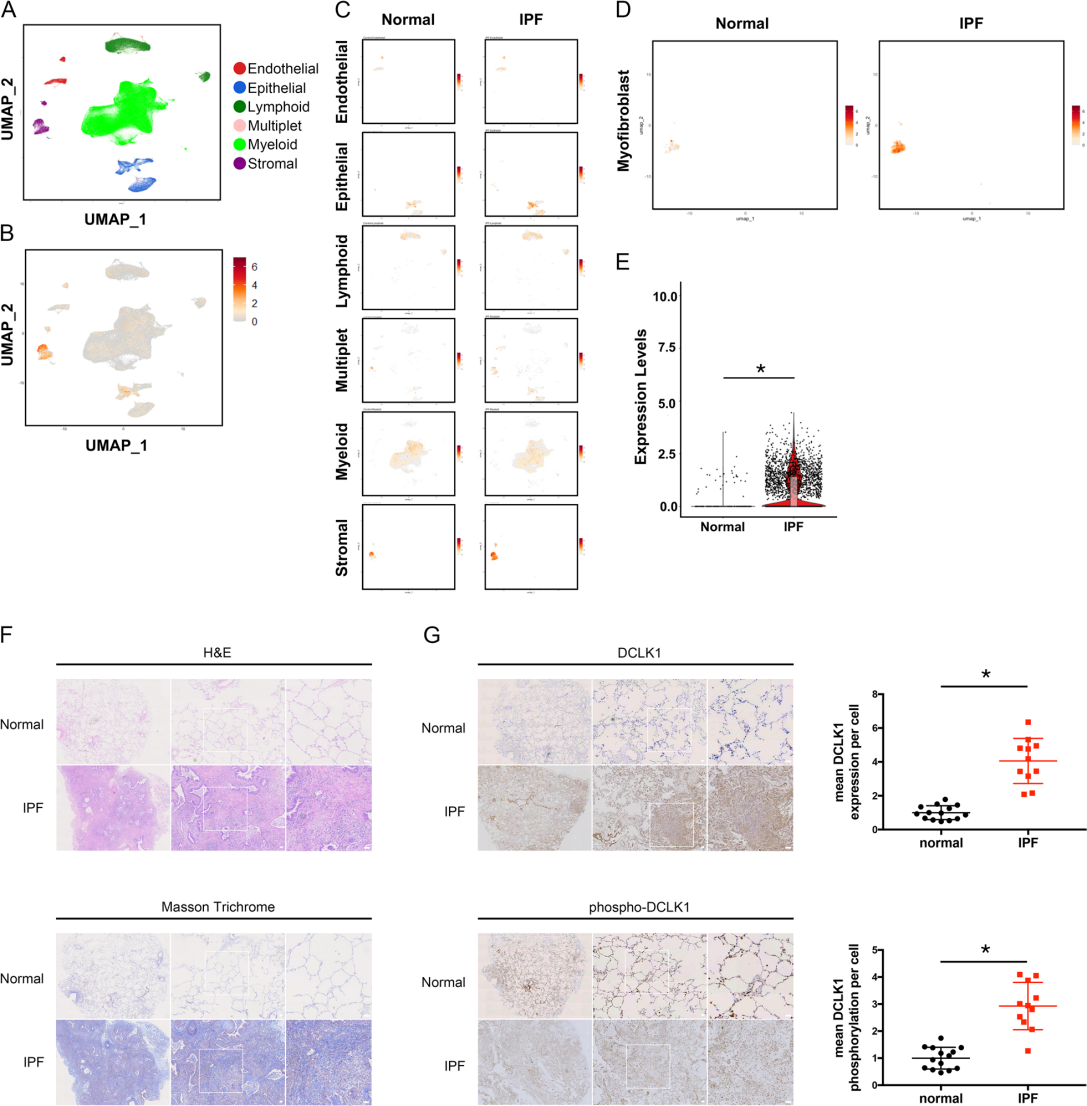
Upregulation of DCLK1 expression and phosphorylation in lung tissue from patients with IPF. **A** UMAP visualization of major lung cell lineages generated from reanalysis of the single-cell RNA sequencing data set GSE136831. **B** Feature plot illustrating the distribution of *DCLK1* expression across all lung cell populations. **C** Feature plots of *DCLK1* expression in major cell lineages from normal lungs and lungs with IPF. **D** Feature plots of the myofibroblast subset derived from the stromal compartment and indicating *DCLK1* expression in normal and IPF samples. **E** Violin plots illustrating log-normalized *DCLK1* expression levels in myofibroblasts from normal lungs and lungs with IPF. Statistical significance was determined using the Wilcoxon rank-sum test. **F** H&E and Masson trichrome staining of lung sections from normal lungs and lungs with IPF (n = 14 control lung tissues and n = 11 IPF lung tissues). **G** Immunohistochemistry results for DCLK1 and phospho-DCLK1 on lung sections from normal lungs and the lungs of patients with IPF (n = 14 control lung tissues and n = 11 IPF lung tissues; 3 nonoverlapping fields analyzed per patient). Original magnifications, 1 × and 4 × . n represents the number of independent individuals per group. Data are presented as means ± SEMs. An unpaired *t* test was used to determine significance relative to normal lung tissue. *p < 0.05 was considered significant. DCLK1, doublecortin-like kinase 1. H&E, hematoxylin and eosin. IPF, idiopathic pulmonary fibrosis. SEM, standard error of the mean. UMAP, uniform manifold approximation and projection

To investigate the role of DCLK1 in IPF pathogenesis, single-cell RNA sequencing analysis was conducted using published data from the GSE136831. The results revealed *DCLK1* expression across multiple lung cell populations, specifically, endothelial, epithelial, lymphoid, myeloid, and stromal cells, with the greatest expression observed in the stromal cells, particularly myofibroblasts (Fig. 1A–E). To confirm the presence of DCLK1 in lungs with IPF, tissue samples were collected from patients with IPF (n = 11) and normal controls (n = 14); the patient demographic characteristics are presented in Table 1. H&E, Masson trichrome, and IHC staining were performed on the serial lung sections. The results of H&E and Masson trichrome staining indicated increased interstitial consolidation and collagen deposition in the lungs with IPF (Fig. 1F). Additionally, DCLK1 and phospho-DCLK1 were upregulated in the IPF lung tissues relative to the control tissues (Fig. 1G, *p < 0.05). These findings support an association between DCLK1 expression and fibrogenesis in the patients with IPF.

**Table 1:** Characteristics of patients with idiopathic pulmonary fibrosis and of controls

Patients								
No	Age (years)	Sex	Diagnosis	FEV1 (%Predicted)	FVC (%Predicted)	FEV1/FVC	DL_CO_ (%Predicted)	Smoker (Current, Ex, Non)
IPF01	57	M	IPF	54.9	51.2	88.1	25.7	Ex
IPF02	68	M	IPF	60.1	52.5	90.1	32.3	Ex
IPF03	48	F	IPF	48.8	50.3	83.7	31.8	Non
IPF04	64	M	IPF	85.4	91.4	74.3	41.5	Non
IPF05	66	F	IPF	47.6	40.7	93.3	50.1	Non
IPF06	43	M	IPF	81.7	77.0	89.1	21.9	Non
IPF07	65	M	IPF	67.5	56.8	95.0	4.9	Current
IPF08	57	M	IPF	38.8	32.0	100.0	39.0	Current
IPF09	57	M	IPF	64.0	59.9	87.9	28.7	Ex
IPF10	68	M	IPF	58.8	52.3	88.6	22.0	Non
IPF11	65	M	IPF	83.0	88.0	70.0	24.0	Ex
Normal01	63	F	Lung tumor	126.8	121.3	83.8	NA	Non
Normal02	73	F	Lung tumor	107	91.2	88.3	NA	Non
Normal03	84	M	Lung tumor/COPD	86	57	98.2	70.3	Current

Commercial slides					
No	Age	Sex	No	Age	Sex
Normal04	26	M	Normal10	48	M
Normal05	50	M	Normal11	38	F
Normal06	49	M	Normal12	25	M
Normal07	40	M	Normal13	22	M
Normal08	55	M	Normal14	35	M
Normal09	35	M			

COPD, chronic obstructive pulmonary disease. DL_CO_, diffusing capacity of the lung for carbon monoxide. F, female. FEV1, forced expiratory volume in 1 s. FVC, forced vital capacity. IPF, idiopathic pulmonary fibrosis. M, male

The IPF group comprised 11 patients. The control group comprised 14 samples, of which 3 were surgically obtained lung tissue samples and 11 were commercially sourced normal lung tissue slides

In contrast to the lung tissues from the IPF group, the commercial slide controls lacked some clinical information (e.g., diagnosis, lung function parameters, DL_CO_, and smoking status)

### DCLK1 knockout attenuates BLM-induced pulmonary fibrosis and decreases fibrotic marker expression

**Fig. 2:**
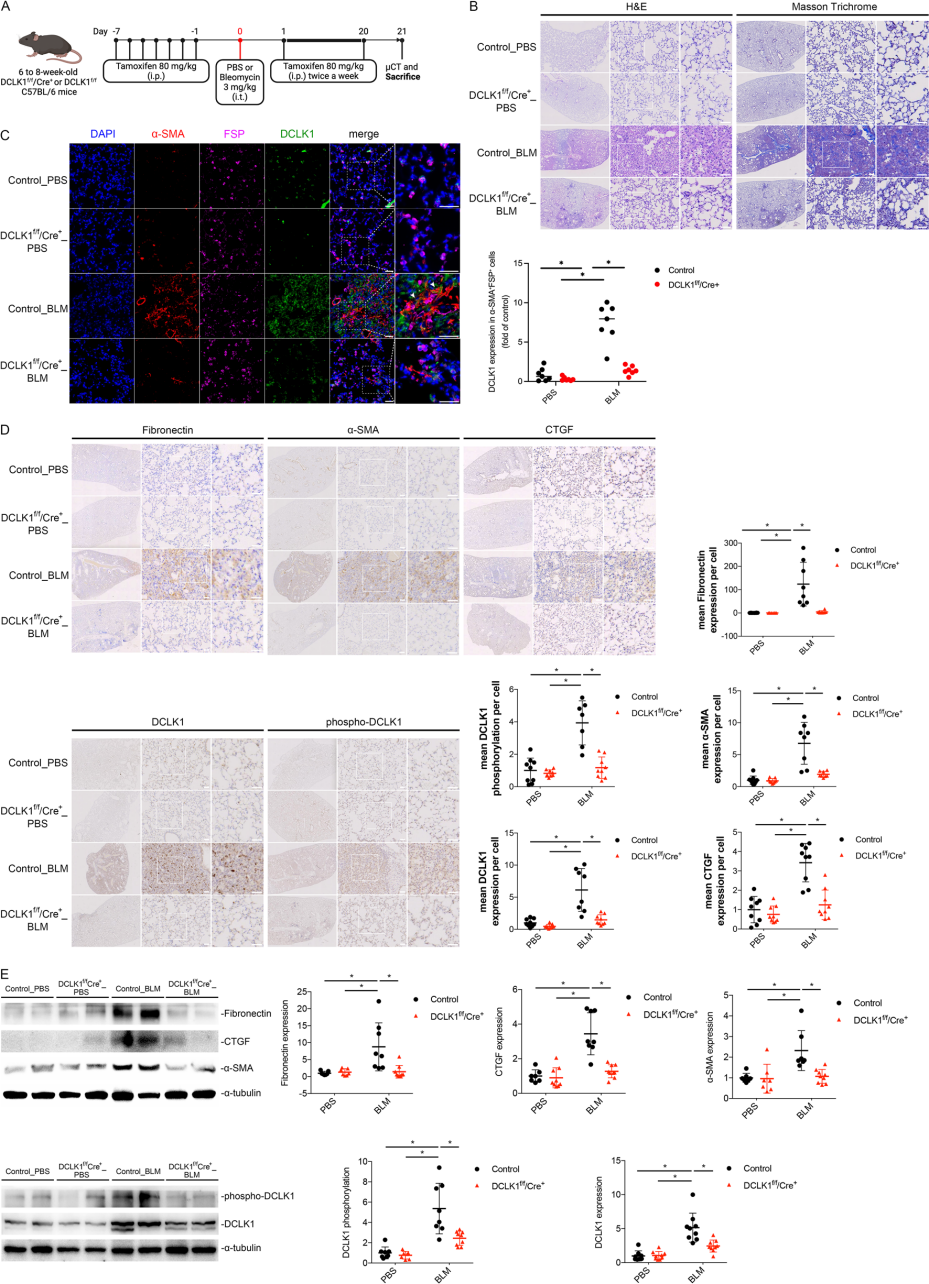
DCLK1 knockout **attenuates** PF and reduces fibrotic protein expression in BLM-treated mice. **A** Experimental design revealing whole-body DCLK1 deletion and BLM intratracheal administration. Tamoxifen (80 mg/kg, i.p.) was administered daily for 7 days before BLM or PBS administration and twice weekly from day 1 to 21 after administration. **B** H&E and Masson trichrome staining of lung tissue (n = 7–9 per group; original magnification, 4 × and 10 ×). **C** Immunofluorescence results for DCLK1 (green), α-SMA (red), FSP (purple), and DAPI (blue; n = 7 per group; original magnification, 20 ×). **D** Immunohistochemistry results for fibronectin, α-SMA, CTGF, DCLK1, and phospho-DCLK1 (n = 7–9 per group; 3 nonoverlapping fields analyzed per animal; original magnification, 4 × and 10 ×). **E** Western blot analysis of lung lysates for fibronectin, α-SMA, CTGF, DCLK1, and phospho-DCLK1 (n = 7–9 per group). n represents the number of independent animals per group. Data are presented as means ± SEMs. A two-way ANOVA and Bonferroni’s multiple comparisons test were used to determine significance relative to BLM-treated control mice. *p < 0.05 was considered significant. α-SMA, alpha–smooth muscle actin. ANOVA, analysis of variance. BLM, bleomycin. CTGF, connective tissue growth factor. DAPI, 4′,6-diamidino-2-phenylindole. DCLK1, doublecortin-like kinase 1. FSP, fibroblast surface protein. H&E, hematoxylin and eosin. i.p., intraperitoneal. i.t., intratracheal. SEM, standard error of the mean

To assess the role of DCLK1 in PF, mice with global *Dclk1* knockout were treated with BLM to induce fibrosis. Tamoxifen (80 mg/kg intraperitoneally) was administered over a period of 7 days and subsequently administered twice a week until the mice were euthanized on day 21 (Fig. 2A). Notably, lung infiltration and collagen deposition were reduced in BLM-treated DCLK1 knockout mice compared with BLM-treated control mice (Fig. 2B). Immunofluorescence analysis showed lower DCLK1⁺/FSP⁺/α-SMA⁺ triple-positive signals in BLM-treated DCLK1 knockout mice compared with BLM-treated control mice (Fig. 2C), consistent with an association between DCLK1 expression and fibroblast-associated activation in fibrotic lungs. Additionally, IHC staining and immunoblots revealed significantly increased expression of DCLK1, phospho-DCLK1, fibronectin, α-SMA, and CTGF in lung tissues from BLM-treated control mice compared with lung tissues from BLM-treated DCLK1 knockout mice (Fig. 2D, 2E, *p < 0.05). These results indicated that knocking out DCLK1 exerted a protective effect against BLM-induced PF.

### DCLK1 knockout mitigates lung dysfunction and morphological damage in mice treated with BLM

**Fig. 3:**
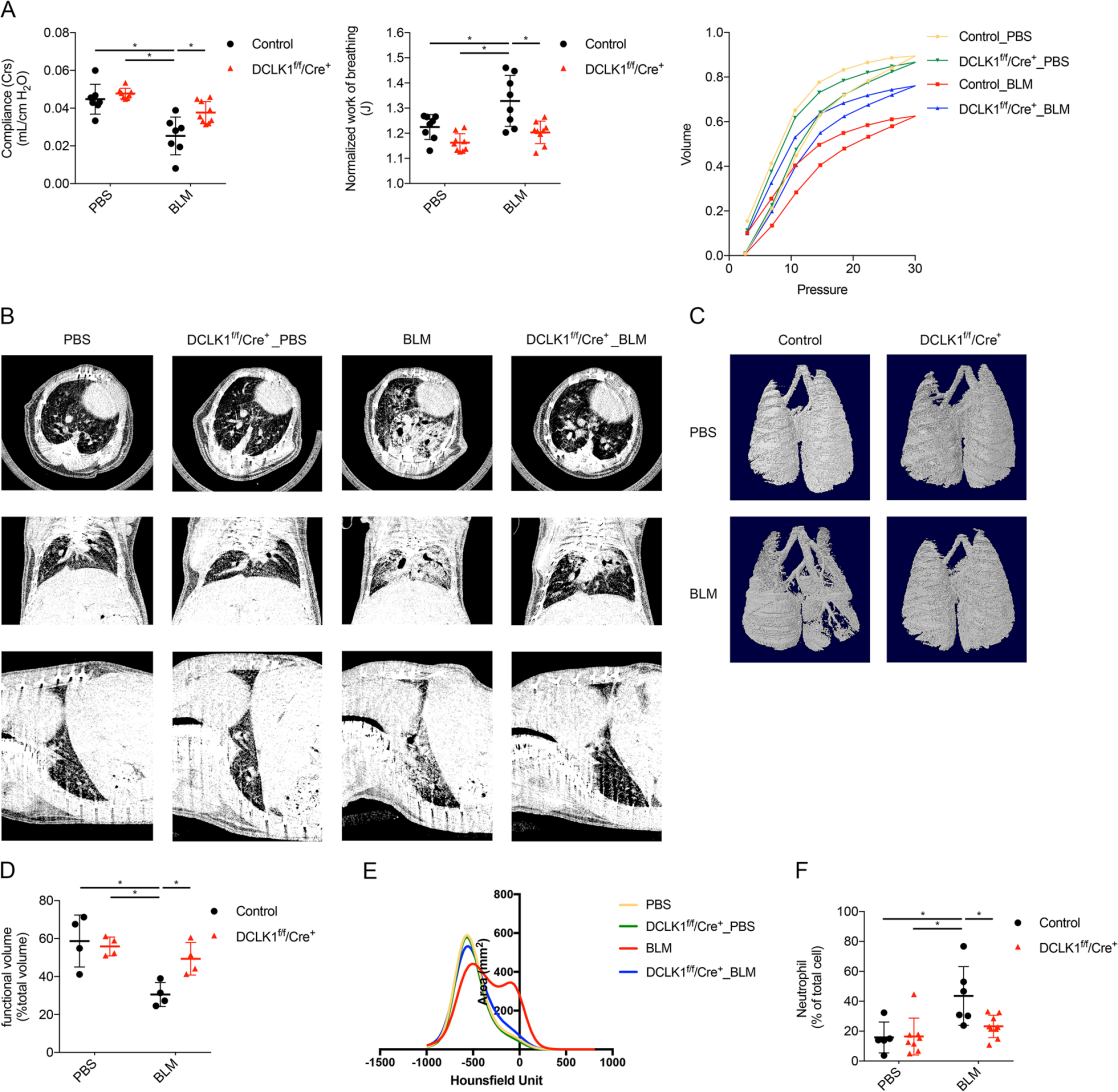
DCLK1 knockout **preserves** lung function, mitigates morphological damage, and decreases inflammation in BLM-treated mice. **A** Lung compliance, normalized work of breathing, and pressure–volume loops measured using the FlexiVent FX system (n = 7–9 per group). **B**–**D** microCT scans, three-dimensional reconstructions, and quantification of lung volume on day 21 (n = 4 per group). **E** Mean HU density histograms derived from segmented lung maps on day 21 (n = 4 per group). **F** Percentage of peripheral blood neutrophils quantified after euthanasia (n = 5–9 per group). n represents the number of independent animals per group. Data are presented as means ± SEMs. A two-way ANOVA and Bonferroni’s multiple comparisons test were used to determine significance relative to BLM-treated control mice. *p < 0.05 was considered significant. ANOVA, analysis of variance. BLM, bleomycin. Crs, lung compliance. HU, Hounsfield unit. microCT, micro computed tomography. nWoB, normalized work of breathing. SEM, standard error of the mean

Lung function impairment and morphological damage are clinical features of IPF. Accordingly, this study conducted lung function tests through the FlexiVent FX system and conducted morphological analyses through in vivo microCT to assess the preclinical effects of DCLK1 knockout. As indicated in Fig. 3A, the respiratory system compliance, pressure–volume loop, and normalized work of breathing were significantly reduced in the BLM-treated control mice, a finding indicative of PF; however, DCLK1 knockout significantly attenuated BLM-induced lung function impairment (*p < 0.05). MicroCT imaging revealed marked parenchymal destruction and volume reduction in the control mouse lungs after BLM was administered. By contrast, DCLK1 knockout attenuated these effects, as indicated by the preserved lung structure and volume in the mice with knocked out DCLK1 (Fig. 3B–D, *p < 0.05). Additionally, mean lung attenuation histograms showed a rightward shift after BLM treatment in wild-type mice, with a greater proportion of voxels above − 500 HU compared to those below this threshold, consistent with increased lung density suggestive of fibrosis. By contrast, DCLK1 knockout preserved lung structure and attenuated the adverse effects of BLM treatment (Fig. 3E). Furthermore, the results of an analysis of peripheral blood samples revealed a significant increase in blood neutrophil percentages in the BLM-treated mice at day 21; this percentage was significantly reduced in the mice with knocked out DCLK1 (Fig. 3F, *p < 0.05). These findings suggest that DCLK1 deletion exerted a protective effect against BLM-induced lung function impairment and morphological damage.

### DCLK1 is associated with TGF-β-induced fibrotic protein expression in NHLFs

**Fig. 4:**
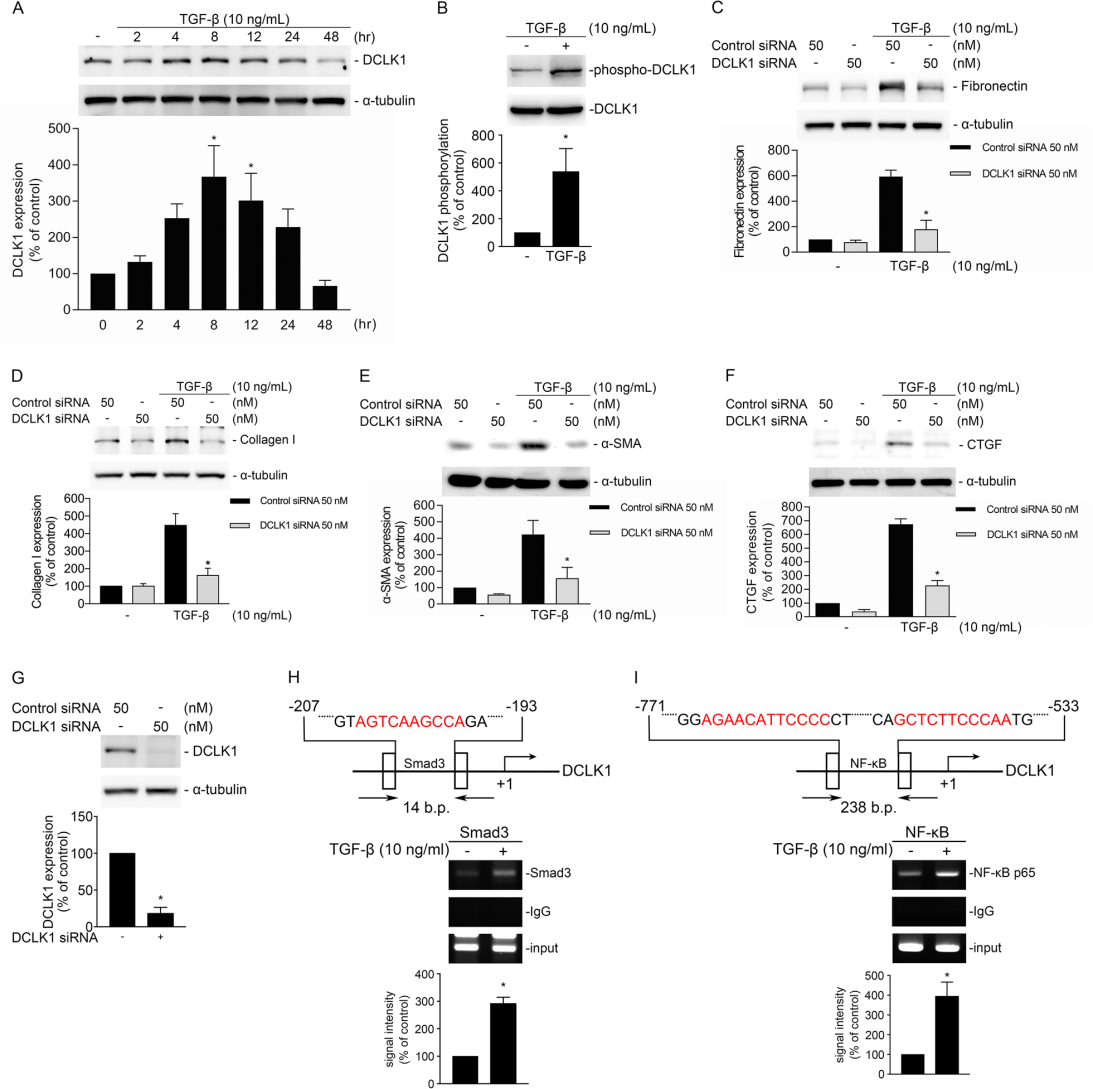
DCLK1 mediates TGF-β-induced fibrotic protein expression in NHLFs. **A**–**B** Western blot analysis of DCLK1 expression and phosphorylation after stimulation with TGF-β (10 ng/mL) for 8 h and 30 min, respectively (n = 4 per experiment). A one-way ANOVA and Dunnett’s test were used to determine significance in **A**, and an unpaired *t* test was used to determine significance in **B**. **C**–**F** Western blot analysis of fibronectin, collagen 1A1, α-SMA, and CTGF after transfection with scrambled siRNA or DCLK1 siRNA and subsequent TGF-β stimulation for 24 h (fibronectin, collagen 1A1, and α-SMA) or 2 h (CTGF; n = 3–5 per experiment). A one-way ANOVA and Dunnett’s test were used to determine significance. **G** Knockdown of DCLK1 confirmed by Western blotting (n = 3). An unpaired *t* test was used to determine significance. **H**–**I** Chromatin immunoprecipitation assays for Smad3 and NF-κB p65 binding to the DCLK1 promoter after 30 min of TGF-β stimulation (n = 3 per experiment). An unpaired *t* test was used to determine significance. n represents the number of independent biological replicates derived from separate cell cultures. Data are presented as means ± SEMs. *p < 0.05 was considered significant. α-SMA, alpha–smooth muscle actin. ANOVA, analysis of variance. CTGF, connective tissue growth factor. DCLK1, doublecortin-like kinase 1. NF-κB, nuclear factor kappa B. NHLF, normal human lung fibroblast. SEM, standard error of the mean. siRNA, small interfering RNA. TGF-β, transforming growth factor beta

To evaluate the role of DCLK1 in TGF-β-induced PF, NHLFs were treated with TGF-β (10 ng/mL), and the expression of fibrotic markers and DCLK1 was assessed. TGF-β induced DCLK1 expression at 8 h and phosphorylation at 30 min (Fig. 4A, B, *p < 0.05). Consistent with the in vivo findings, DCLK1 siRNA-transfected NHLFs exhibited significantly reduced expression of fibronectin, collagen 1A1, α-SMA, and CTGF after TGF-β treatment (Fig. 4C–G, *p < 0.05). ChIP assays showed enrichment of Smad3 and NF-κB p65 at the DCLK1 promoter region after TGF-β stimulation (Fig. 4H,I), supporting a link between TGF-β-responsive Smad3/NF-κB p65 signaling and DCLK1 expression.

### Akt/DCLK1/Smad3 signaling is associated with TGF-β-induced CTGF expression in NHLFs

**Fig. 5:**
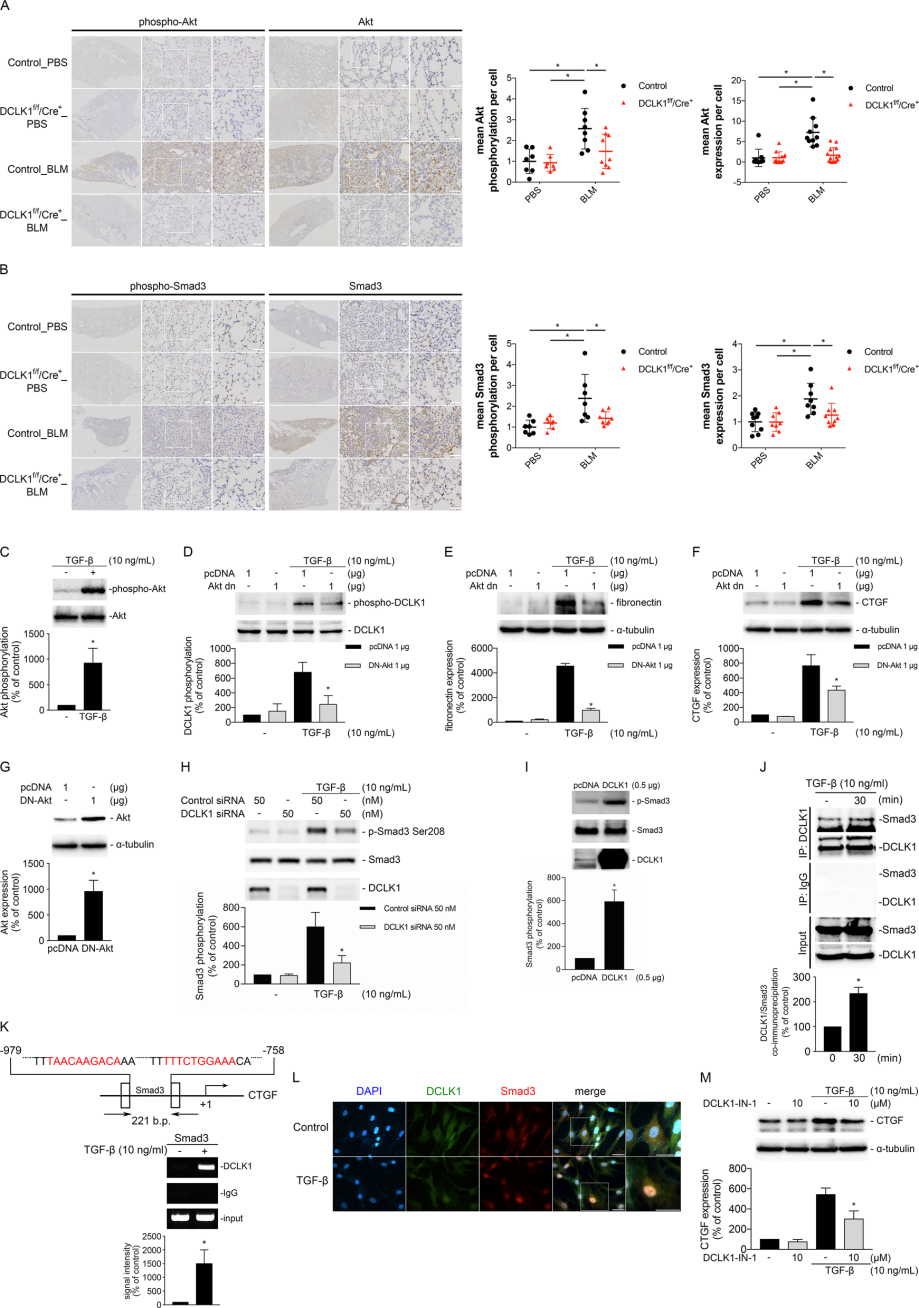
Akt/DCLK1/Smad3 signaling is associated with TGF-β-induced CTGF expression in NHLFs. **A**–**B** Immunohistochemistry results for phospho-Akt, Akt, phospho-Smad3, and Smad3 in lung tissues from PBS-treated and BLM-treated control mice with or without DCLK1 deletion (n = 7–9 per group; 3 nonoverlapping fields analyzed per animal; original magnification, 4 × and 10 ×). A two-way ANOVA and Bonferroni’s multiple comparisons test were used to determine significance. **C** Western blot analysis of phospho-Akt in NHLFs stimulated with TGF-β (10 ng/mL) for 30 min (n = 3). An unpaired *t* test was used to determine significance. **D–F** Western blot analysis of phospho-DCLK1, fibronectin, and CTGF in NHLFs transfected with pcDNA or DN-Akt, followed by TGF-β stimulation for 30 min (DCLK1), 2 h (CTGF), or 24 h (fibronectin; n = 3 per experiment). A one-way ANOVA and Dunnett’s test were used to determine significance. **G** Western blot confirmation of Akt inhibition (n = 3). An unpaired *t* test was used to determine significance. **H** Western blot analysis of phospho-Smad3 in NHLFs transfected with DCLK1 siRNA or scrambled siRNA and stimulated with TGF-β for 30 min (n = 4). A one-way ANOVA and Dunnett’s test were used to determine significance. **I** Western blot analysis of Smad3 phosphorylation in NHLFs overexpressing DCLK1 and stimulated with TGF-β for 30 min (n = 4). An unpaired *t* test was used to determine significance. **J** Coimmunoprecipitation of DCLK1 and Smad3 after TGF-β stimulation for 30 min (n = 6). An unpaired *t* test was used to determine significance. **K** Chromatin immunoprecipitation analysis using a DCLK1 antibody targeting the Smad3-binding region of the CTGF promoter after TGF-β stimulation for 30 min (n = 3). An unpaired *t* test was used to determine significance. **L** Immunofluorescence results for DCLK1 (green), Smad3 (red), and DAPI (blue) in NHLFs after TGF-β stimulation for 30 min (n = 4; original magnification, 40 ×). **M** Western blot analysis of CTGF expression in NHLFs pretreated with DCLK1-IN-1 (10 μM, 30 min) before TGF-β stimulation for 2 h (n = 3). A one-way ANOVA and Dunnett’s test were used to determine significance. n represents the number of independent animals per group (in vivo) or independent biological replicates derived from separate cell cultures (in vitro). Data are presented as means ± SEMs. *p < 0.05 was considered significant. ANOVA, analysis of variance. BLM, bleomycin. Co-IP, coimmunoprecipitation. CTGF, connective tissue growth factor. DAPI, 4′,6-diamidino-2-phenylindole. DCLK1, doublecortin-like kinase 1. DN-Akt, dominant negative Akt. DCLK1-IN-1, doublecortin-like kinase 1 inhibitor 1. NHLF, normal human lung fibroblast. PBS, phosphate-buffered saline. SEM, standard error of the mean. siRNA, small interfering RNA. TGF-β, transforming growth factor beta

We examined the role of DCLK1 in TGF-β-induced Akt and Smad3 activation and CTGF expression in NHLFs. In the mouse model, phosphorylation and expression of Akt and Smad3 were significantly increased in the lung interstitial regions of the mice administered BLM but reduced in the mice with DCLK1 knockout (Fig. 5A, B, *p < 0.05). In the NHLFs, TGF-β significantly induced Akt phosphorylation (Fig. 5C, *p < 0.05), and DN-Akt transfection significantly reduced TGF-β-induced DCLK1 phosphorylation and the expression of fibronectin and CTGF (Fig. 5D–G, *p < 0.05). In addition, DCLK1 siRNA transfection reduced TGF-β-induced Smad3 phosphorylation (Fig. 5H, *p < 0.05), whereas DCLK1 overexpression enhanced TGF-β-induced Smad3 phosphorylation (Fig. 5I, *p < 0.05). Coimmunoprecipitation analysis showed an association between DCLK1 and Smad3 (Fig. 5J, *p < 0.05). Moreover, ChIP using a DCLK1 antibody showed enrichment at the Smad3-binding region of the CTGF promoter (Fig. 5K, *p < 0.05). Together, these findings are consistent with DCLK1–Smad3-associated signaling linked to CTGF expression. Immunofluorescence analysis further showed overlapping nuclear signals of DCLK1 and Smad3 after 30 min of TGF-β treatment (Fig. 5L). Pretreatment with the DCLK1 inhibitor DCLK1-IN-1 reduced TGF-β-induced CTGF expression (Fig. 5M, *p < 0.05). These results supported an association between Akt/DCLK1/Smad3 signaling and TGF-β-induced CTGF expression in NHLFs.

### DCLK1-IN-1 attenuates fibrotic progression, preserves lung function, and reduces morphological damage and inflammation in BLM-treated mice

**Fig. 6:**
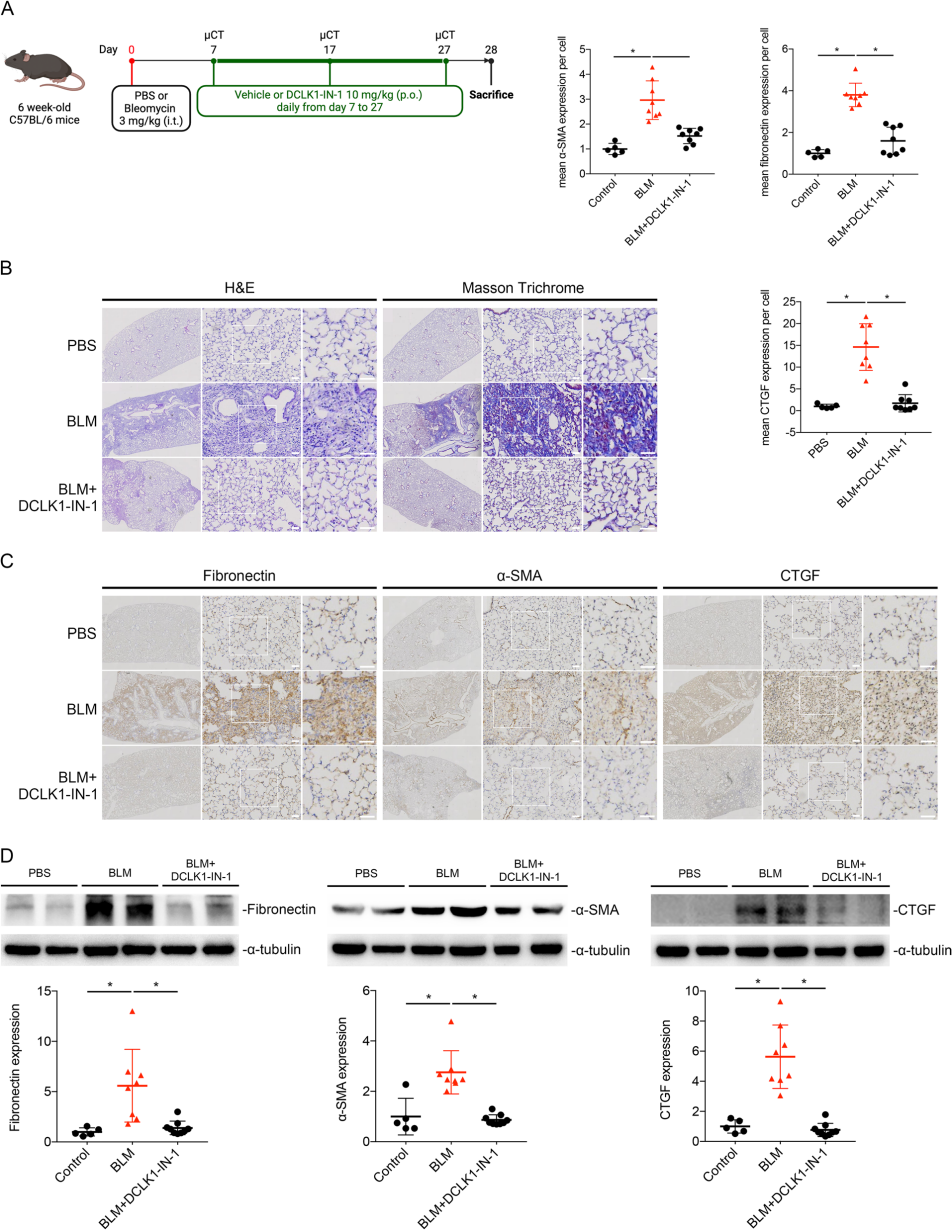
DCLK1-IN-1 attenuates fibrotic progression and reduces fibrotic protein expression in BLM-treated mice. **A** Experimental design for DCLK1-IN-1 oral gavage (10 mg/kg daily) starting on day 7 after BLM administration until endpoint. **B** Results of H&E and Masson trichrome staining of lung tissues (n = 5–8 per group; original magnification, 4 × and 10 ×). **C** Immunohistochemistry results for fibronectin, α-SMA, and CTGF (n = 5–8 per group; 3 nonoverlapping fields analyzed per animal; original magnification, 4 × and 10 ×). **D** Western blot analysis of lung lysates for fibronectin, α-SMA, and CTGF (n = 5–8 per group). n represents the number of independent animals per group. Data are presented as means ± SEMs. A one-way ANOVA and Dunnett’s test were used to determine significance relative to BLM-treated control mice. *p < 0.05 was considered significant. α-SMA, alpha–smooth muscle actin; ANOVA, analysis of variance. BLM, bleomycin. CTGF, connective tissue growth factor. DCLK1-IN-1, doublecortin-like kinase 1 inhibitor 1. H&E, hematoxylin and eosin. PF, pulmonary fibrosis. SEM, standard error of the mean

To evaluate whether pharmacological inhibition of DCLK1 attenuates fibrotic progression, the DCLK1 inhibitor DCLK1-IN-1 (10 mg/kg) was administered by oral gavage to BLM-treated mice. DCLK1-IN-1 was administered daily from day 7 after BLM administration until euthanasia on day 28 (Fig. 6A). Histological analysis showed reduced fibrotic progression in the (BLM + DCLK1-IN-1)-treated mice compared with the BLM-only group (Fig. 6B). Immunohistochemical analysis demonstrated reduced expression of fibrotic markers, including fibronectin, α-SMA, and CTGF, in lung sections from (BLM + DCLK1-IN-1)-treated mice compared with BLM-only mice (Fig. 6C). Similarly, lung lysates from the (BLM + DCLK1-IN-1)-treated mice exhibited significantly lower fibronectin, α-SMA, and CTGF expression (Fig. 6D, *p < 0.05).

**Fig. 7:**
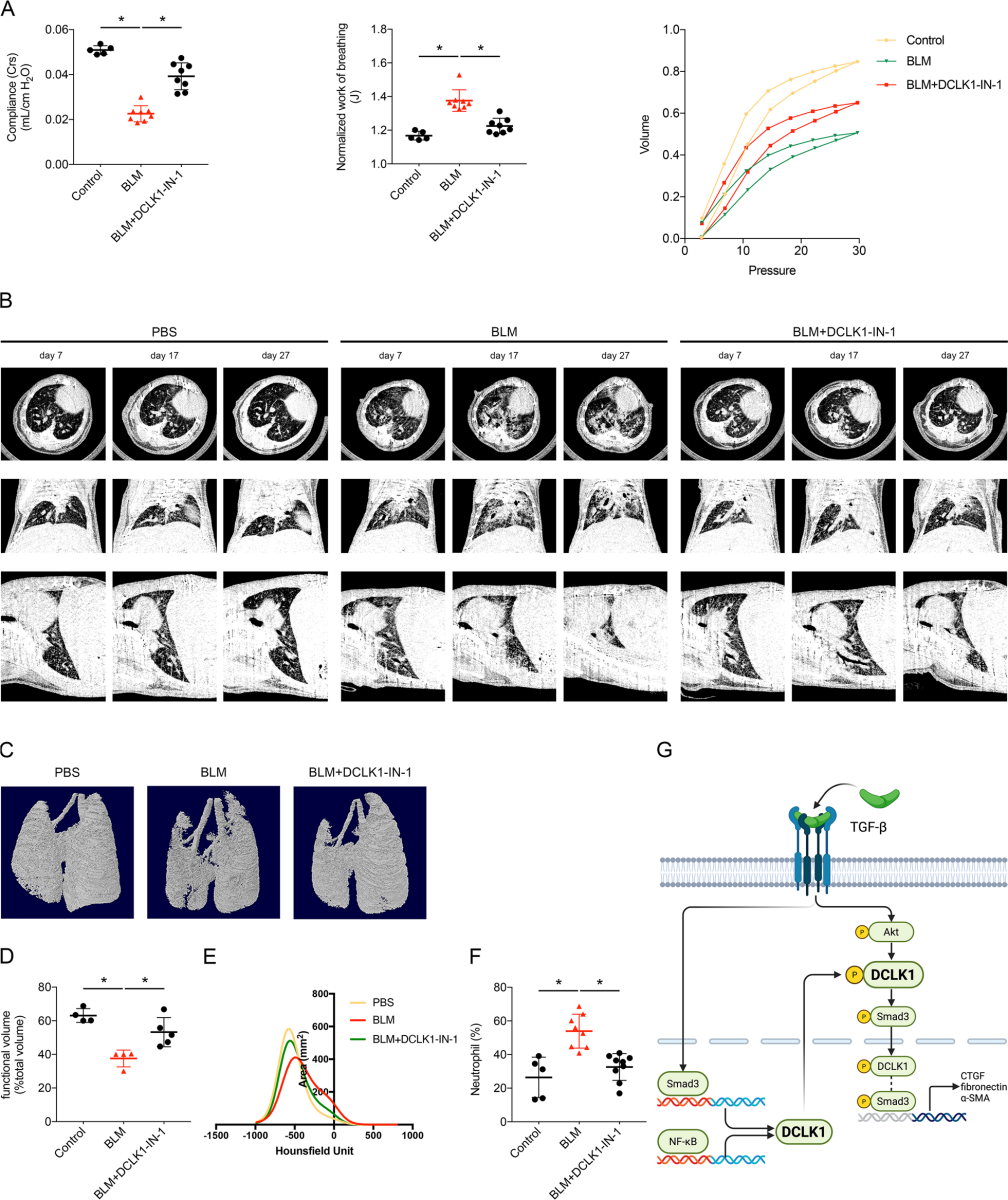
DCLK1-IN-1 preserves lung function, reduces morphological damage, and decreases inflammation in BLM-treated mice. **A** Lung compliance, normalized work of breathing, and pressure–volume loops measured by using the FlexiVent FX system (n = 5–8 per group). **B**–**D** MicroCT scans, three-dimensional reconstructions, and quantification of lung volume on day 28 (n = 4–5 per group). **E** Mean HU density histograms derived from segmented lung maps at day 28 (n = 4–5 per group). **F** Percentage of peripheral blood neutrophils quantified after euthanasia (n = 5–9 per group). **G** Schematic of signaling pathways indicating TGF-β–induced DCLK1 expression through Smad3 and NF-κB and proposed model linking TGF-β-induced DCLK1 expression with Akt/DCLK1/Smad3-associated signaling and profibrotic marker expression. n represents the number of independent animals per group. Data are presented as means ± SEMs. A one-way ANOVA and Dunnett’s test were used to determine significance compared with BLM-treated control mice. *p < 0.05 was considered significant. α-SMA, alpha–smooth muscle actin. ANOVA, analysis of variance. BLM, bleomycin. Crs, lung compliance. CTGF, connective tissue growth factor. DCLK1-IN-1, doublecortin-like kinase 1 inhibitor 1. HU, Hounsfield unit. microCT, micro computed tomography. NF-κB, nuclear factor kappa B. nWoB, normalized work of breathing. SEM, standard error of the mean. TGF-β, transforming growth factor beta

DCLK1-IN-1 administration preserved pulmonary compliance and reduced the normalized work of breathing in the BLM-treated mice, with corresponding improvements in pressure–volume loop patterns (Fig. 7A, *p < 0.05). MicroCT imaging on days 7, 17, and 27 after the administration of BLM was used to monitor lung morphology and revealed more severe fibrotic changes in the mice treated with BLM only than in those treated with DCLK1-IN-1 (Fig. 7B, 7D, *p < 0.05). The three-dimensional lung structures at the endpoint were subsequently reconstructed using microCT images (Fig. 7C). In lung attenuation histograms, the proportion of voxels with attenuation values > − 500 HU was lower in the BLM + DCLK1-IN-1 group than in the BLM group (Fig. 7E). Additionally, treatment with DCLK1-IN-1 significantly reduced blood neutrophil percentages in the BLM-treated mice at day 28 (Fig. 7F, *p < 0.05). These results suggest that DCLK1-IN-1 attenuated fibrotic progression and preserved lung function in the BLM-induced pulmonary fibrosis model.

## Discussion

This study demonstrated that DCLK1 may contribute to PF pathogenesis. Specifically, DCLK1 was upregulated in the lung interstitium of patients with IPF and in BLM-induced fibrotic mouse lungs. Mechanistic investigations suggested that DCLK1 is associated with profibrotic signaling in lung fibroblasts. DCLK1 altered Smad3 phosphorylation and was associated with Smad3, while ChIP findings were consistent with DCLK1–Smad3-associated signaling linked to CTGF expression. Together, these findings support a potential role of DCLK1 in fibroblast activation and fibrotic progression. In addition, DCLK1 expression was induced by TGF-β through both Smad-dependent and Smad-independent pathways. In vivo, global *Dclk1* deletion attenuated BLM-induced PF and respiratory dysfunction in mice. Oral administration of DCLK1-IN-1 in BLM-treated fibrotic mouse lungs also slowed fibrosis progression and preserved lung function. These findings indicate that DCLK1 is associated with TGF-β-related profibrotic signaling and PF progression and suggest that DCLK1 warrants further investigation as a potential therapeutic target in fibrotic lung disease. A proposed model linking DCLK1 to fibrotic progression is depicted in Fig. 7G.

DCLK1 is a multifunctional kinase implicated in various pulmonary diseases that is expressed across several lung cell types, specifically, epithelial, endothelial, immune, and mesenchymal cells. In severe asthma, thrombin induces DCLK1 activation and RhoA signaling, leading to nuclear translocation of Yes-associated protein, which associates with NF-κB p65 to promote IL-8/CXCL8 transcription in human bronchial epithelial cells [26]. In acute respiratory distress syndrome (ARDS), DCLK1 is involved in alveolar epithelial type II (AECII) cell differentiation after lipopolysaccharide (LPS)-induced lung injury; in early ARDS, DCLK1-mediated Yes-associated protein signaling promotes lung epithelial repair, and this pattern is reversed in the later stages of the condition [27]. DCLK1 has also been implicated in immune regulation, including in cytokine expression and immune cell dysregulation in SARS-CoV-2-induced sepsis, in which its inhibition attenuates caspase-1/IL-1β signaling and mitigates cytokine secretion and the activation of inflammatory pathways in SARS-CoV-2-infected cells, normalizing cytokine/chemokine profiles [11]. Furthermore, DCLK1 is a marker of epithelial stem-like cells and may be involved in their repair [7]. The potential involvement of DCLK1 in lung cancer progression and immune modulation indicates that it affects several cellular compartments in lung pathology [28]. Notably, our findings indicate that in PF, DCLK1 was not restricted to a single cell type but was expressed across lung cell populations. However, we focused on fibroblast-associated signaling because fibroblasts are responsible for extracellular matrix production and fibrotic progression. We demonstrated that DCLK1 knockout preserved lung function in a BLM-induced PF model. These observations suggest that DCLK1 is associated with fibroblast-related fibrotic progression and warrants further investigation as a potential therapeutic target in fibrotic lung disease.

TGF-β signaling regulates numerous cellular processes through both canonical and noncanonical pathways [29]. The canonical pathway is principally mediated by Smad proteins and begins when TGF-β binds to its receptors, which facilitates phosphorylation and activation of Smad2 and Smad3 [30]. These receptor-activated Smads oligomerize with Smad4 and translocate to the nucleus to regulate gene transcription [31]. Moreover, Smad3 often functions within multiprotein complexes to modulate gene transcription. For example, Smad3 forms a complex with Gli2 to promote PTHrP expression in breast cancer metastasis [32]; it also associates with NCID to induce Hes1 and Foxp3 expression, influencing cell cycle regulation during fibrogenesis [33]. In addition to the canonical route, TGF-β activates noncanonical pathways involving molecules such as Akt and NF-κB (p65), which contribute to cell survival, proliferation, and differentiation [34]. Akt influences downstream effectors such as mTOR and GSK-3β through PI3K signaling, affecting metabolism and growth [35], whereas NF-κB p65 is activated by TRAF6 and TAK1 to promote inflammation and cell survival [36, 37]. The present study suggested that TGF-β induced DCLK1 expression in PF through both canonical and noncanonical pathways. Furthermore, DCLK1 intersects the TGF-β signaling network through its association with Smad3, with findings consistent with DCLK1–Smad3-associated signaling linked to CTGF expression. The interactions between these pathways reveal the complexity of the TGF-β signaling that maintains cellular homeostasis and promotes fibrogenesis.

Notably, a study on single-cell transcriptomic profiling of fibrotic skin revealed substantial heterogeneity in fibroblasts and distinct profibrotic subpopulations that may promote disease progression [38]. Another study identified specialized subsets of cells such as immune-modulating fibroblasts and tissue-remodeling myofibroblasts [39]. The heterogeneity reported in the aforementioned studies suggests that fibroblasts contribute to fibrosis through several mechanisms other than simple matrix deposition; one study reported that these mechanisms comprise secretion of profibrotic cytokines, modulation of immune cell recruitment, and maintenance of a persistent fibrogenic microenvironment [40]. In the present study, DCLK1 expression was observed across multiple lung cell populations, including fibroblast-associated compartments. Our immunofluorescence analysis showed partial co-localization of DCLK1 with FSP⁺/α-SMA⁺ fibroblast-associated markers in fibrotic lungs, supporting a potential link between DCLK1 expression and fibroblast-associated activation. Future studies using cell type–specific approaches and higher-resolution single-cell analyses are needed to determine whether DCLK1 expression marks functionally distinct cell subsets, including fibroblast subsets, and whether these subsets contribute to disease severity and progression. Such studies may help clarify whether DCLK1-targeted strategies can modulate pathogenic fibrotic progression while preserving homeostatic or reparative cell populations.

Only two drugs approved by the US Food and Drug Administration are available to treat IPF: nintedanib (Ofev) and pirfenidone (Esbriet) [41]. Nintedanib is a small-molecule tyrosine kinase inhibitor of vascular endothelial growth factor, fibroblast growth factor, and platelet-derived growth factor receptors, whereas pirfenidone inhibits TGF-β production and activity [42]. Although both drugs slow fibrosis progression, they do not reverse or cure the condition. In addition, research has demonstrated that a DCLK1 inhibitor can reduce IkappaB kinase-beta phosphorylation in macrophages after LPS stimulation, inhibiting inflammatory signaling and mitigating LPS-induced lung injury and sepsis [43]. A phase 3 randomized clinical trial evaluating pamrevlumab, a monoclonal antibody against CTGF, demonstrated that treatment did not substantially mitigate declines in lung function or improve clinical outcomes compared with placebo in patients with IPF [44]. These findings suggest that targeting downstream fibrotic end products alone may be insufficient to halt disease progression, highlighting the need for alternative strategies that target upstream signaling regulators or involve combination therapies [45]. In the present study, DCLK1 knockout and inhibition attenuated BLM-induced fibrosis and reduced TGF-β-induced fibrotic protein expression, supporting further investigation of DCLK1 as a potential therapeutic target in PF. Additional preclinical studies are required to clarify the safety, efficacy, and translational relevance of DCLK1-targeted approaches in fibrotic lung diseases.

This study has several limitations. First, DCLK1 is expressed across multiple lung cell populations, including fibroblasts, immune cells, AECII cells, and endothelial cells, as indicated by the single-cell RNA sequencing and IF analysis results. This wide expression of DCLK1 suggests that it may affect fibrogenesis through multiple cellular sources [26, 27]. In addition, the Pgk1-Cre/ERT2 system used in the present study mediated a global, tamoxifen-inducible deletion of *Dclk1* rather than targeting specific cell types; consequently, the contributions of multiple lung cell populations to fibrogenesis could not be fully distinguished. Further research is required to clarify the cell-type–specific effects of DCLK1 inhibition and the role of such inhibition in fibrogenesis. Second, this study examined heterogeneous human control lung tissues, specifically tumor-adjacent samples, lungs with COPD, and commercially sourced normal lung tissue. Because lung biopsies from truly healthy individuals are rarely available for ethical reasons, these sources are commonly used as substitutes for nonfibrotic controls in studies of human PF. Nevertheless, potential confounding effects could not be fully excluded. Third, the mechanisms underlying the association between DCLK1 and Smad3, such as subcellular interactions and the regulation of DCLK1 expression in IPF, BLM-induced PF, and TGF-β signaling, have yet to be clarified. Fourth, although our study focused on mitigating fibrosis progression, whether DCLK1 inhibition promotes fibrosis resolution and lung tissue regeneration remains unclear. Nevertheless, this question must be addressed to develop therapies that halt or reverse established fibrosis. Finally, because DCLK1 is involved in cancer and other systemic processes, potential off-target or systemic effects of long-term DCLK1 inhibition must be carefully evaluated in preclinical studies.

## Conclusions

This study explored the role of DCLK1 in IPF and BLM-induced PF. Notably, DCLK1 knockout and inhibition attenuated both morphological and functional alterations in the lungs caused by BLM. TGF-β induced DCLK1 expression through Smad-dependent and Smad-independent pathways and was associated with increased expression of fibrotic markers, particularly CTGF, linked to Akt/DCLK1/Smad3 signaling. In addition, DCLK1 was associated with Smad3, with findings consistent with DCLK1–Smad3-associated signaling linked to fibrotic marker expression. Overall, our findings suggest that DCLK1 may represent a potential therapeutic target in PF that warrants further investigation.

## Data Availability

The single-cell RNA sequencing data of IPF and healthy control tissue were obtained from the publicly available GEO data set GSE136831 (https://www.ncbi.nlm.nih.gov/geo/query/acc.cgi?acc=GSE136831). Additional data are available from the corresponding author upon reasonable request.

## Abbreviations

AECII: Alveolar Epithelial Type II Cell
α-SMA: Alpha–Smooth Muscle Actin
ARDS: Acute Respiratory Distress Syndrome
BLM: Bleomycin
ChIP: Chromatin Immunoprecipitation
COPD: Chronic Obstructive Pulmonary Disease
Crs: Compliance
CTGF: Connective Tissue Growth Factor
DCLK1: Doublecortin-Like Kinase 1
HU: Hounsfield Unit
i.p.: Intraperitoneal
IPF: Idiopathic Pulmonary Fibrosis
i.t.: Intratracheal
LPS: Lipopolysaccharide
microCT: Microcomputed Tomography
NF-κB: Nuclear Factor Kappa B
nWoB: Normalized Work of Breathing
PF: Pulmonary Fibrosis
TGF-β: Transforming Growth Factor Beta
